# Dual-Energy Computed Tomography-Guided Assessment of Visceral Adiposity and Its Correlation With Lipid Function Test: A Retrospective Study

**DOI:** 10.7759/cureus.69618

**Published:** 2024-09-17

**Authors:** Ajina Sam, Afrin Banu Kaja Mohideen, Mohamed Asif Syed Buhari, Karthik Krishna Ramakrishnan, Shree Haritha P

**Affiliations:** 1 Radiodiagnosis, Saveetha Medical College and Hospital, Saveetha Institute of Medical and Technical Sciences, Saveetha University, Chennai, IND; 2 Medical Imaging Technology, Saveetha College of Allied Health Sciences, Saveetha Institute of Medical and Technical Sciences, Saveetha University, Chennai, IND

**Keywords:** cholesterol, dual-energy computed tomography, dual-energy ct (dect), lipid function test, metabolic disorders, visceral adiposity

## Abstract

Introduction

Obesity, a prevalent global health concern, is associated with various chronic conditions, including cardiovascular diseases and metabolic syndrome. Visceral adiposity, the accumulation of fat around internal organs, has a more significant impact on metabolic health compared to subcutaneous fat. Accurate assessment of visceral fat is critical for predicting metabolic risks. Dual-Energy Computed Tomography (DECT) is emerging as an effective tool for quantifying visceral adiposity, allowing for enhanced tissue differentiation. This study aims to assess visceral adiposity using DECT and explore its correlation with lipid function tests, including cholesterol and triglyceride levels, in a cohort of patients.

Materials and methods

This retrospective observational study was conducted in the Department of Radiology at Saveetha Medical College and Hospital, Chennai. Data from 100 patients aged 25 to 75 years with a BMI of 25 kg/m² or higher, were analyzed. DECT scans were performed using a Siemens SOMATOM go.Top 128-slice CT scanner (Siemens, Munich) to quantify visceral fat, particularly mesenteric fat. Lipid function tests were conducted to measure total cholesterol, LDL cholesterol, HDL cholesterol, and triglycerides. Pearson’s correlation coefficient was used to analyze the relationship between visceral fat volume and lipid profile components.

Results

The study found significant correlations between visceral adiposity and lipid profile components. Total Visceral Fat Area (VFA) volume positively correlated with total cholesterol (r = 0.65, p < 0.01), LDL cholesterol (r = 0.58, p < 0.01), and triglycerides (r = 0.52, p < 0.05). An inverse relationship was observed between VFA volume and HDL cholesterol (r = -0.48, p < 0.05). Regression analysis confirmed that VFA volume is an independent predictor of these lipid levels after adjusting for age, gender, and BMI. The study also reported the prevalence of hepatomegaly in 11 (36.6%) cases and fatty liver in nine (30%) cases in the study population, underscoring the metabolic implications of visceral fat accumulation.

Conclusion

This study highlights the significant role of visceral adiposity in influencing lipid metabolism and associated cardiovascular risks. DECT proved to be a precise and reliable tool for assessing visceral fat and its metabolic implications. The findings suggest that increased visceral fat is associated with adverse lipid profiles, contributing to a higher risk of metabolic disorders. These results emphasize the need for incorporating advanced imaging techniques like DECT in clinical practice for better risk stratification and personalized treatment strategies in patients with obesity and related metabolic conditions.

## Introduction

Obesity, a prevalent global health concern, is linked to multiple chronic conditions, including cardiovascular diseases, type 2 diabetes mellitus, and metabolic syndrome. Within the spectrum of adiposity, visceral adiposity, characterized by the accumulation of fat within the abdominal cavity around internal organs, stands out due to its particularly harmful effects. Unlike subcutaneous fat, which is located just beneath the skin, visceral fat is more metabolically active and has a pronounced association with adverse metabolic outcomes, such as insulin resistance, dyslipidemia, and hypertension. These metabolic disturbances contribute significantly to the overall morbidity and mortality associated with obesity, making the assessment of visceral adiposity crucial for understanding and managing these risks [1,2]. 

Given the critical role of visceral adiposity in metabolic health, the accurate assessment of visceral fat is necessary for predicting and managing associated metabolic risks. Traditional methods, such as anthropometric measurements and bioelectrical impedance analysis, often fall short in providing the precision and reliability needed for effective clinical decision-making. Advanced imaging techniques, such as computed tomography (CT) and magnetic resonance imaging (MRI), have emerged as the gold standards for assessing visceral fat. Among these, dual-energy computed tomography (DECT) is particularly noteworthy. DECT utilizes two different X-ray energy levels to enhance material differentiation and tissue characterization, allowing for precise quantification of visceral adiposity [3].

The primary aim of this study is to utilize DECT to accurately assess visceral adiposity, with a specific focus on quantifying mesenteric fat volume in a cohort of patients with obesity. This study seeks to explore the correlation between visceral adiposity and lipid function test results, including total cholesterol, low-density lipoprotein (LDL) cholesterol, high-density lipoprotein (HDL) cholesterol, and triglycerides. By examining these relationships, the study aims to understand how variations in visceral fat volumes influence lipid metabolism and contribute to dyslipidemia, assess the correlation of visceral adiposity with other key metabolic indicators such as BMI and fatty liver disease, and evaluate the effectiveness of DECT in clinical practice for managing obesity-related metabolic disorders [4].

Through these objectives, the study aspires to contribute to the development of more effective strategies for risk stratification, monitoring, and personalized treatment of patients with obesity and related metabolic disorders. By providing a detailed analysis of the relationship between visceral fat and metabolic health, this research could serve as a pivotal guide for future studies and clinical practices, ultimately leading to better management and outcomes for patients affected by obesity.

## Materials and methods

Study design and population

This study was conducted as a retrospective observational analysis at the Department of Radiology, Saveetha Medical College and Hospital, Chennai. The study involved the analysis of data from 100 patients who were referred for investigational procedures. The patients were selected based on specific inclusion criteria: adults aged 25 to 75 years with a body mass index (BMI) of 25 kg/m² or higher. Exclusion criteria included patients on lipid-lowering medications, pregnant or breastfeeding women, individuals with conditions that significantly affect body fat distribution (such as Cushing's syndrome), and those with incomplete imaging or lipid profile data.

Imaging protocol

Each patient underwent a DECT scan using a Siemens SOMATOM go.Top® 128-slice CT scanner (Siemens, Munich). The scans were performed with the patients in a supine position, and simultaneous acquisition of low-energy (80 kVp) and high-energy (140 kVp) X-ray beams was conducted during a single breath-hold to minimize motion artifacts [5,6]. The imaging covered the abdominal region from the diaphragm to the pelvic brim.

Visceral fat quantification

**Figure 1:**
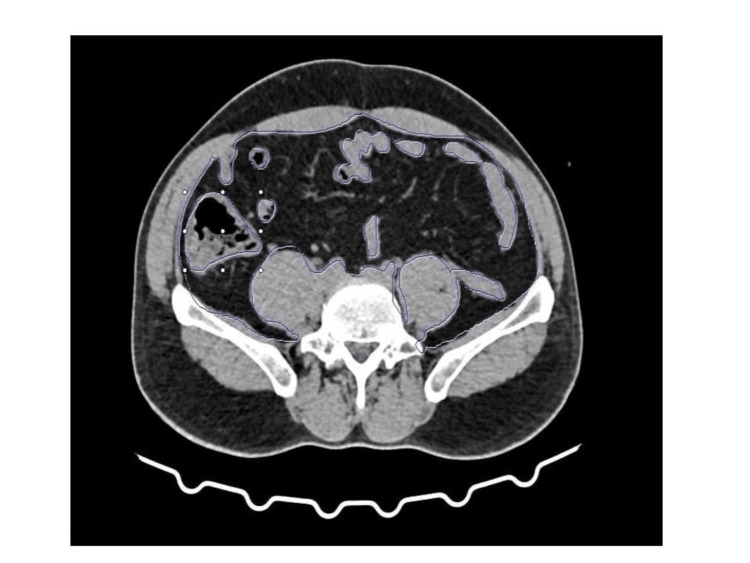
Delineating the visceral fat Delineating the visceral fat using the region-growing tool, ensuring that the segmentation process captured all relevant adipose tissue without including surrounding structures.

**Figure 2:**
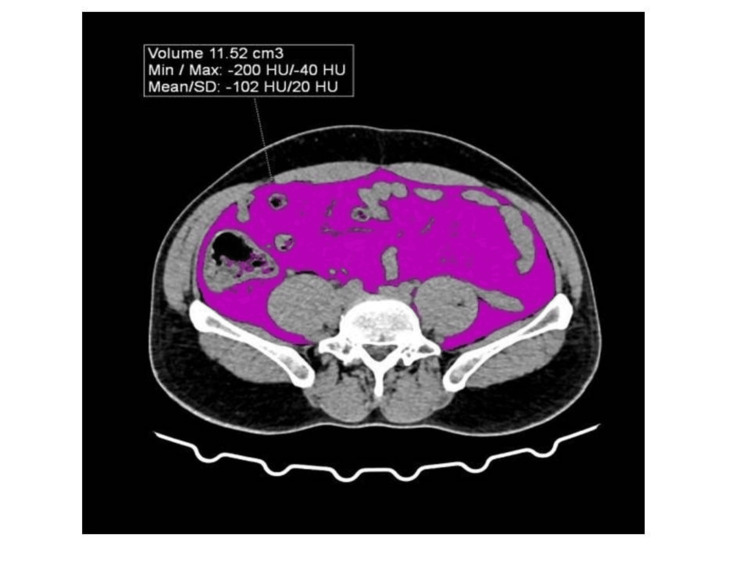
Quantification of volume of visceral adiposity

Visceral fat quantification involved loading the CT images into the Syngovia software (Siemens Healtheeniers, USA) for analysis. The abdominal region was segmented, and the region of interest (ROI) was identified specifically around the mesenteric fat. The region-growing tool was utilized to accurately delineate the visceral fat, ensuring that the segmentation process captured all relevant adipose tissue without including surrounding structures, as shown in Figure 1. The volume of visceral adiposity was then quantified, as shown in Figure 2.

Lipid function tests

Lipid function tests were conducted for all patients, including measurements of total cholesterol, low-density lipoprotein (LDL) cholesterol, high-density lipoprotein (HDL) cholesterol, and triglycerides [7]. These measurements provided the necessary data to analyze the correlation between visceral fat volume and lipid metabolism.

Statistical analysis

The relationship between the quantified visceral fat and the lipid profile results was analyzed using descriptive statistics to summarize the study population's characteristics (Table 1). Pearson’s correlation coefficient was employed to determine the relationship between visceral adiposity (measured as Total Visceral Fat Area volume) and lipid profile components (total cholesterol, low-density lipoprotein (LDL) cholesterol, high-density lipoprotein (HDL) cholesterol, and triglycerides) [8,9]. Linear regression analysis was conducted to adjust for potential confounders, including age, gender, and BMI, and to confirm visceral fat as an independent predictor of lipid levels.

**Table 1:** Descriptive statistics VFA: Visceral Fat Area; LDL: Low-density lipoprotein; HDL: High-density lipoprotein.

Variable	Mean	Std. Dev.	Min	Max
Age (years)	52.8	12.4	26	74
BMI (kg/m^2^)	26.4	3.5	20.3	34.4
Total VFA volume (cc)	9286.5	4672.1	2670	20026
Total cholesterol (mg/dL)	170.3	54.8	83	351
HDL cholesterol (mg/dL)	33.7	13.1	13	75
LDL cholesterol (mg/dL)	110.1	63.5	33	288.2
Triglycerides (mg/dL)	187.8	110.5	53	646
Total cholesterol/HDL Ratio	5.3	1.9	2	10.6

## Results

Correlation analysis

The correlation analysis demonstrated significant relationships between visceral adiposity, as measured by total Visceral Fat Area (VFA) volume, and various lipid profile components. As summarized in Table 2, a significant positive correlation was found between total VFA volume and total cholesterol (r = 0.65, p < 0.01), indicating that as visceral fat increases, total cholesterol levels tend to rise. A significant positive correlation was also observed between total VFA volume and LDL cholesterol (r = 0.58, p < 0.01), suggesting that higher visceral fat is associated with elevated LDL cholesterol levels. Similarly, the correlation between total VFA volume and triglycerides (r = 0.52, p < 0.05) indicates that increased visceral fat is linked to higher triglyceride levels. In contrast, a significant negative correlation was found between total VFA volume and HDL cholesterol (r = -0.48, p < 0.05). This inverse relationship suggests that higher visceral fat is associated with lower levels of HDL cholesterol, which is known for its protective role in cardiovascular health.

**Table 2:** Correlation analysis VFA: Visceral Fat Area; LDL: Low-density lipoprotein; HDL: High-density lipoprotein. Significant p-values are denoted by '*', where significance is considered at p < 0.05 and ** for p < 0.01.

Variable Pair	Correlation Coefficient (r)	p-value
Total VFA volume vs. total cholesterol	0.65	<0.01**
Total VFA volume vs. HDL cholesterol	-0.48	<0.05*
Total VFA volume vs. LDL cholesterol	0.58	<0.01**
Total VFA volume vs. triglycerides	0.52	<0.05*

Regression analysis

Total cholesterol: As shown in Table 3, total VFA volume is a significant independent predictor of total cholesterol levels (β = 0.0078, p < 0.01). This suggests that for every unit increase in VFA volume, there is a corresponding increase in total cholesterol, even after adjusting for potential confounders.

**Table 3:** Regression analysis for total cholesterol VFA: Visceral Fat Area. Significant p-values are denoted by '*', where significance is considered at p < 0.05 and ** for p < 0.01

Parameter	Coefficient	Std. Error	t-statistic	p-value
Total cholesterol (Constant)	97.81	21.53	4.54	<0.001**
Total VFA volume	0.0078	0.0023	3.39	<0.01**

LDL cholesterol: The regression analysis for LDL cholesterol, as presented in Table 4, shows that total VFA volume is also a significant independent predictor (β = 0.0081, p < 0.001). This supports the finding that increased visceral fat is strongly associated with higher LDL cholesterol levels, a key risk factor for cardiovascular disease.

**Table 4:** Regression analysis for LDL cholesterol VFA: Visceral fat area; LDL: Low-density lipoprotein. Significant p-values are denoted by '*', where significance is considered at p < 0.05 and ** for p < 0.01

Parameter	Coefficient	Std. Error	t-statistic	p-value
LDL cholesterol (Constant)	35.41	16.98	2.09	<0.05*
Total VFA volume	0.0081	0.0018	4.50	<0.001**

HDL cholesterol: For HDL cholesterol, as detailed in Table 5, the regression analysis indicates that total VFA volume is a significant independent predictor, but with a negative coefficient (β = -0.0012, p < 0.05). This highlights that as visceral fat increases, HDL cholesterol levels decrease, which is detrimental to cardiovascular health.

**Table 5:** Regression analysis for HDL cholesterol VFA: Visceral fat area; LDL: High-density lipoprotein. Significant p-values are denoted by '*', where significance is considered at p < 0.05 and ** for p < 0.01.

Parameter	Coefficient	Std. Error	t-statistic	p-value
HDL cholesterol (Constant)	45.23	5.93	7.63	<0.001**
Total VFA volume	-0.0012	0.0006	-2.00	<0.05*

Triglycerides: The regression analysis for triglycerides, shown in Table 6, indicates that total VFA volume is a significant independent predictor (β = 0.0084, p < 0.05). This confirms that higher visceral fat is associated with elevated triglyceride levels, another important marker of metabolic and cardiovascular risk.

**Table 6:** Regression analysis for triglycerides VFA: Visceral fat area. Significant p-values are denoted by '*', where significance is considered at p < 0.05 and ** for p < 0.01

Parameter	Coefficient	Std. Error	t-statistic	p-value
Triglycerides (Constant)	123.62	29.72	4.16	<0.001**
Total VFA volume	0.0084	0.0031	2.71	<0.05*

Hepatomegaly and fatty liver distribution

The study also examined the distribution of hepatomegaly and fatty liver among the participants. As shown in Table 7, hepatomegaly (measured in CT as cranio-caudal length, more than 16.5 cm in males and 16 cm in males) was present in 11 (36.6%) of the patients, indicating a substantial prevalence of liver enlargement in the study population, which is often associated with visceral fat accumulation and metabolic disorders.

**Table 7:** Hepatomegaly distribution

Hepatomegaly	No. of Cases	Percentage
Present	11	36.6%
Absent	19	63.3%
Total	30	100%

Similarly, the distribution of fatty liver was assessed, and as detailed in Table 8, fatty liver (liver attenuation lower than 40 HU in CT) was present in 9 (30%) of the patients, further underscoring the connection between visceral fat and liver-related metabolic conditions.

**Table 8:** Fatty liver distribution

Fatty Liver	No. of Cases	Percentage
Present	9	30%
Absent	21	70%
Total	30	100%

These results highlight the critical role of visceral adiposity in influencing lipid metabolism and associated cardiovascular risk. The significant correlations and regression findings, supported by the data presented in Tables 2 to 8, indicate that total VFA volume is an independent predictor of lipid levels, even after accounting for age, gender, and BMI. Furthermore, the prevalence of hepatomegaly and fatty liver in the study population emphasizes the metabolic implications of increased visceral fat.

## Discussion

The findings from this study highlight the crucial role of visceral adiposity in the metabolic derangements commonly associated with obesity. The use of dual-energy computed tomography (DECT) has been demonstrated to provide a highly accurate and reliable method for quantifying visceral fat, marking a significant advancement over traditional single-energy CT and other imaging modalities like MRI [10,11]. DECT's ability to differentiate tissue types based on their absorption of different X-ray energy levels allows for precise measurements of visceral fat, which is critical for understanding its impact on lipid metabolism and associated metabolic risks [3,7].

The strong correlation observed in this study between increased visceral adiposity and elevated levels of LDL cholesterol and triglycerides, along with reduced levels of HDL cholesterol, aligns with the current understanding of visceral fat as a metabolically active tissue that significantly contributes to dyslipidemia. This relationship is well-supported in the literature, particularly in studies by Després and colleagues, who have consistently linked visceral fat to adverse metabolic profiles, including insulin resistance and cardiovascular risk factors [4,12].

Compared to previous studies that utilized traditional imaging techniques, DECT offers enhanced tissue differentiation, resulting in stronger correlations between visceral adiposity and lipid profiles. For example, while Palmas et al. documented limitations in single-energy CT's ability to accurately assess adipose tissue, DECT in this study provided more precise measurements, thereby demonstrating its superior capability in correlating visceral fat with metabolic markers [5].

Furthermore, DECT has shown considerable promise in other clinical applications, particularly in vascular imaging. For instance, DECT can produce low-energy virtual monoenergetic images that enhance iodine attenuation, improving the visualization of small vessels and lesions. This capability not only aids in assessing vascular conditions but also offers insights into the perfusion of tissues, which could be particularly useful in evaluating the metabolic activity of adipose tissues. This feature contrasts with the limitations of MRI and single-energy CT, which may not provide the same level of detail in certain clinical scenarios [13,14].

Additionally, the study underscores the potential of DECT in reducing artifacts and improving image quality, particularly in patients with metal implants or those undergoing complex imaging studies. The ability to generate virtual non-contrast images further reduces patient radiation exposure, a significant advantage in longitudinal studies where repeated imaging is required. This aligns with findings by Borga et al., who emphasized the importance of advanced imaging techniques in body composition assessment and highlighted the challenges associated with traditional methods [13].

While the findings of this study are consistent with previous research validating DECT's accuracy in quantifying adipose tissue compared to MRI, it is essential to consider the cost and accessibility of this technology. For instance, Machann et al., demonstrated that while MRI remains the gold standard for certain aspects of adipose tissue quantification, DECT offers several practical advantages in clinical settings, including lower radiation doses and improved image clarity. However, these benefits may be offset by the higher costs and limited availability of DECT, particularly in resource-constrained environments [6,14].

This study contributes to the growing body of evidence supporting the use of DECT in the precise quantification of visceral adiposity and its correlation with lipid function tests. The insights gained from this research could inform more effective risk stratification and personalized treatment strategies for patients with obesity and related metabolic disorders. Future research should focus on expanding these findings in larger, more diverse populations and exploring the potential of DECT in other areas of metabolic and cardiovascular health. Additionally, comparative studies between DECT, MRI, and other emerging imaging modalities would further clarify the relative strengths and limitations of each, guiding their optimal use in clinical practice [9,15].

Despite the strengths of this study, several limitations should be acknowledged. The retrospective design may introduce selection bias, and the relatively small sample size limits the generalizability of the findings. Additionally, while DECT offers enhanced tissue differentiation and accurate quantification of visceral adiposity, its availability and cost may restrict its widespread use in clinical practice, particularly in resource-limited settings. The study did not include a direct comparison with other imaging modalities, such as MRI, which could provide a more comprehensive understanding of DECT's relative advantages and limitations. Furthermore, technical factors, such as patient body habitus and the potential for imaging artifacts, may affect the accuracy of DECT measurements, and these were not fully addressed in this study.

## Conclusions

In conclusion, this study demonstrated a significant correlation between visceral adiposity, as measured by DECT, and lipid function test results, reinforcing the critical role of visceral fat in the pathogenesis of metabolic disorders. The findings highlight the utility of DECT as a precise and reliable tool for assessing visceral fat and its metabolic implications. By providing detailed insights into the relationship between visceral adiposity and lipid metabolism, this study contributes to the development of more effective strategies for risk stratification, monitoring, and personalized treatment of patients with obesity and related metabolic conditions. Future research should focus on expanding these findings in larger cohorts and exploring the potential of DECT in other areas of metabolic and cardiovascular health.
